# Methods for conducting trends analysis: roadmap for comparing outcomes from three national HIV Population-based household surveys in Kenya (2007, 2012, and 2018)

**DOI:** 10.1186/s12889-022-13633-8

**Published:** 2022-07-13

**Authors:** Thomas Achia, Ismael Flores Cervantes, Paul Stupp, Paul Musingila, Jacques Muthusi, Anthony Waruru, Mary Schmitz, Megan Bronson, Gregory Chang, John Bore, Leonard Kingwara, Samuel Mwalili, James Muttunga, Joshua Gitonga, Kevin M. De Cock, Peter Young

**Affiliations:** 1 Division of Global HIV & TB, Center for Global Health, US Centers for Disease Control and Prevention, Nairobi, Kenya; 2 Westat, Rockville, MD USA; 3 Division of Global HIV and TB, Centers for Disease Control and Prevention, Atlanta, GA USA; 4 Division of Global HIV and TB, Center for Global Health, US Centers for Disease Control and Prevention, Kisumu, Kenya; 5 Kenya National Bureau of Statistics, Nairobi, Kenya; 6 National AIDS and STI Control Program, Nairobi, Kenya; 7 Department of Statistics and Actuarial Science, Jomo Kenyatta University, Juja, Kenya; 8 Kenya Medical Research Institute, Nairobi, Kenya; 9 National AIDS Control Council, Nairobi, Kenya

**Keywords:** HIV, Trends, Survey design, Stratification, Survey weights, Clustering, Multistage sampling

## Abstract

**Background:**

For assessing the HIV epidemic in Kenya, a series of independent HIV indicator household-based surveys of similar design can be used to investigate the trends in key indicators relevant to HIV prevention and control and to describe geographic and sociodemographic disparities, assess the impact of interventions, and develop strategies. We developed methods and tools to facilitate a robust analysis of trends across three national household-based surveys conducted in Kenya in 2007, 2012, and 2018.

**Methods:**

We used data from the 2007 and 2012 Kenya AIDS Indicator surveys (KAIS 2007 and KAIS 2012) and the 2018 Kenya Population-based HIV Impact Assessment (KENPHIA 2018). To assess the design and other variables of interest from each study, variables were recoded to ensure that they had equivalent meanings across the three surveys. After assessing weighting procedures for comparability, we used the KAIS 2012 nonresponse weighting procedure to revise normalized KENPHIA weights. Analyses were restricted to geographic areas covered by all three surveys. The revised analysis files were then merged into a single file for pooled analysis. We assessed distributions of age, sex, household wealth, and urban/rural status to identify unexpected changes between surveys.

To demonstrate how a trend analysis can be carried out, we used continuous, binary, and time-to-event variables as examples. Specifically, temporal trends in age at first sex and having received an HIV test in the last 12 months were used to demonstrate the proposed analytical approach. These were assessed with respondent-specific variables (age, sex, level of education, and marital status) and household variables (place of residence and wealth index). All analyses were conducted in SAS 9.4, but analysis files were created in Stata and R format to support additional analyses.

**Results:**

This study demonstrates trends in selected indicators to illustrate the approach that can be used in similar settings. The incidence of early sexual debut decreased from 11.63 (95% CI: 10.95–12.34) per 1,000 person-years at risk in 2007 to 10.45 (95% CI: 9.75–11.2) per 1,000 person-years at risk in 2012 and to 9.58 (95% CI: 9.08–10.1) per 1,000 person-years at risk in 2018. HIV-testing rates increased from 12.6% (95% CI: 11.6%–13.6%) in 2007 to 56.1% (95% CI: 54.6%–57.6%) in 2012 but decreased slightly to 55.6% [95% CI: 54.6%–56.6%) in 2018. The decrease in incidence of early sexual debut could be convincingly demonstrated between 2007 and 2012 but not between 2012 and 2018. Similarly, there was virtually no difference between HIV Testing rates in 2012 and 2018.

**Conclusions:**

Our approach can be used to support trend comparisons for variables in HIV surveys in low-income settings. Independent national household surveys can be assessed for comparability, adjusted as appropriate, and used to estimate trends in key indicators. Analyzing trends over time can not only provide insights into Kenya’s progress toward HIV epidemic control but also identify gaps.

**Supplementary Information:**

The online version contains supplementary material available at 10.1186/s12889-022-13633-8.

## Introduction

In 2017, despite the rapid increase in antiretroviral therapy (ART) use over the previous two decades and the corresponding decline in mortality, approximately one-third of people living with HIV in East and Southern Africa and less than half of those living with HIV in West and Central Africa were not receiving any life-saving treatment [1, 2]. By 2017, HIV/AIDS was a major cause of death in sub-Saharan Africa (SSA), where 71% of all people living with HIV resided. Globally, 75% of HIV-related deaths and 65% of all new HIV infections occurred in SSA [3–5].

Against this background, it is important to assess whether interventions over the last two to three decades have decreased HIV incidence and to identify geographic regions and sociodemographic groups with high HIV prevalence [3, 6]. HIV data obtained from national population-based surveys play an important role in monitoring the HIV epidemic and response in the general population. These surveys estimate incidence, prevalence, and various parameters related to the HIV pandemic in high-HIV-prevalence countries. These surveys were designed to monitor progress toward ending the AIDS epidemic [6–8]. Additionally, they were designed to monitor the UNAIDS 90–90-90 targets by the year 2020: 90% of all HIV-positive people know their HIV status; of these, 90% are receiving sustained ART; and of these, 90% have achieved viral load suppression [9–11]. These surveys have also been used to describe associations between high-risk behavior and HIV status and to assess HIV prevention, care, and treatment services.

Unlike in high-income countries where longitudinal studies provide nationally representative trend estimates for health outcomes, for example, the National Health and Nutrition Examination Survey [12, 13], HIV surveys in low-income countries and high-prevalence settings are generally cross-sectional and are independently implemented approximately once every 5 years. Therefore, it is important to develop methods that can be used to assess trends across independent surveys for countries interested in employing similar techniques. We used Kenya to showcase this approach as there had been several HIV population-based surveys conducted, with varying sampling and survey weighting considerations, in the past two decades. Such methods must account for differences in survey design, weighting, coverage, and indicator definitions. Over the past two decades, five national population-based surveys [14–18] have included HIV testing and HIV modules in their algorithms in Kenya.

We present methods that can be used to assess temporal trends in outcome variables of interest as a means to answer such questions as: “Has HIV risk behavior significantly declined over time in Kenya, and if so, in which demographic groups or regions?” and “Has access to HIV testing services increased over time in Kenya?” Our tools also can help HIV programs appropriately analyze trends in recent population-based HIV surveys in Kenya and provide guidance regarding appropriate statistical comparisons between surveys, including tests for trends. These suggestions may also serve as a roadmap for other cross-survey comparison analyses applicable to other countries or indicators. The methods presented here are being utilized to examine trends in specific indicators of interest in other KENPHIA-focused studies. Therefore, the programmatic implications of selected trends comparison presented in this study are not discussed.

## Methods

### Harmonization of survey datasets

#### Analysis approach

First, we reviewed survey design documents to describe the survey design and weighting procedures used for all three surveys. We compared sampling design and survey weighting procedures across surveys to identify differences that could potentially influence comparisons. We developed an analysis strategy to both facilitate comparisons and minimize the influence of differences in survey design or weighting procedures on comparisons between survey estimates. Once we chose a weighting approach, we developed a list of variables to extract and harmonize across surveys based on perceived importance, availability, and consistency of definitions across surveys. Once extracted, the weighted estimates of these variables were assessed for consistency across surveys. Finally, we used selected variables to identify and describe appropriate statistical methods for comparisons and trend analysis.

#### Data extraction and manipulation

We reviewed data dictionaries and other survey documentation to identify relevant survey design and analysis variables pertaining to HIV biomarkers and behavioral and demographic variables across the three surveys for inclusion in the analysis.

#### Survey design

These surveys were originally designed to provide data used by various stakeholders to monitor Kenya’s population and HIV-related health outcomes. This section briefly summarized the survey design and weighing approaches used in the surveys. All three surveys utilized two-stage stratified, cluster sampling designs based on the National Sample Survey and Evaluation Programme (NASSEP) household-based sample frames created by the Kenya National Bureau of Statistics and revised after each decennial population census.

KAIS 2007 was the first AIDS Indicator Survey conducted in Kenya to monitor progress on key indicators in the national HIV prevention, care, and treatment programs [16]. The survey was designed to obtain a nationally representative sample of persons aged 15–64 years and to provide estimates of HIV-related outcomes stratified by urban/rural residence and the 8 provinces. The first stage included a selection of 415 clusters (70% rural and 30% urban) from the NASSEP IV (based on the 1999 census); the second stage included selecting a sample of 25 households within each cluster.

KAIS 2012 selected 372 clusters from NASSEP V (based on the 2009 census) using a systematic random sampling method. KAIS 2012 sampled 9,300 households within 9 of the 10 National AIDS and STI Control Programme (NASCOP) programmatic regions: Nairobi, Central, Coast, Eastern North, Eastern South, Nyanza, Upper Rift, Lower Rift, and Western regions, designated as either urban or rural. The sampling frame was not available for the North-Eastern region at the time of the survey, and this region (and hence seven NASCOP regions) was excluded from the survey. The target population was persons aged 18 months–64 years. Half of the households were targeted for children aged 18 months–14 years. The survey was designed to provide estimates of HIV-related outcomes for adults aged 15–64 years stratified by urban/rural area and the nine included NASCOP regions.

Like KAIS 2012, KENPHIA 2018 also was based on NASSEP V. KENPHIA was a cross-sectional, household-based survey conducted among persons aged 0–64 years in 800 clusters from 96 urban/rural county strata covering the entire household population of Kenya. In 2012, following the promulgation of the 2010 Constitution of Kenya, these counties became the geographical units of devolved government in place of districts. Survey data collection was conducted from June 2018 to February 2019. Of the 34,610 persons targeted by the survey, 27,897 were adults aged 15–64 years, and 6,713 were children aged 0–14 years. One in three households were targeted for the inclusion of children. The survey was designed to provide estimates for adults aged 15–64 years for all 47 counties in Kenya.

Each of these studies were carried out in accordance with the Helsinki Declaration.

Table 1 presents detailed summaries of the three surveys.

**Table 1:** Summary of survey designs and HIV testing for KAIS 2007, KAIS 2012 and KENPHIA 2018

Survey	Survey dates	Design	Interviews	Sample for HIV module	Target population	Sampling frame
KAIS 2007	August-December, 2007	Two-stage stratified cluster sample, where the first stage included selection of 415 clusters from the NASSEP IV, stratified by district and residency, and for urban areas, by socio-economic status, and the second stage included selection of 25 households within each selected cluster	9,691 households, 17,940 adults and adolescents aged 15–64 years	Collected 15,853 blood specimens from 9,049 women and 6,804 men	De facto household resident adults and adolescents aged 15–64 years	National Sample Survey and Evaluation Programme (NASSEP) IV developed from 1999 census; comprised of 1,800 clusters
KAIS 2012	October 29, 2012 to February 3, 2013	Two-stage stratified cluster sample, where the first stage included selection of 372 clusters from NASSEP V, stratified by county and residency, and the second stage included selection of 25 households within each selected cluster. For the child sample, every other household was selected	8,035 households, 13,720 adults and adolescents aged 15–64 years and 1,661 children aged 10–14	Collected 15,966 blood specimens from 4,832 women and 6,785 men; 2,131 girls and 2208 boys aged 18 months to 14 years	De facto household resident children aged 18 months to 14 years; adults and adolescents aged 15 to 64 years	NASSEP V developed from 2009 census; comprised of 5,360 clusters
KENPHIA 2018	June 2018 to February 2019	Two-stage, stratified cluster sample design where the first stage included selection of 800 clusters from NASSEP V and the second stage included selection of 25 households within each selected cluster. For child sample, every third household was selected	16,918 households, 30,384 adults and adolescents aged 15–64 years and 2,687 children aged 10–14	Collected 35,610 blood specimens from 11,726 men and 16,019 women; 4090 boys and 3775 girls	De facto household resident children aged 0–14 years; Adults and adolescents aged 15 to 64 years	NASSEP V

*Abbreviations: NASSEP* National Sample Survey and Evaluation Programme, *KAIS* Kenya AIDS Indicator survey, *KENPHIA* Kenya Population-based HIV Impact Assessment

### Weighting process

#### Stratification

The KAIS 2007 design was stratified by district and residency (urban/rural). Urban areas were further stratified by socioeconomic status. Both KAIS 2012 and KENPHIA designs were stratified by county and residency. Household nonresponse adjustments in KAIS 2007 were computed by province and residency, whereas in KAIS 2012, they were computed by NASCOP region and residency, resulting in the following nineteen design strata: Nairobi (Urban), Central (Urban/Rural), Nyanza (Urban/Rural), North Rift (Urban/Rural), South Rift (Urban/Rural), Eastern North (Urban/Rural), Eastern South (Urban/Rural), Western (Urban/Rural), and Coast (Urban/Rural). In KENPHIA, household nonresponse adjustments were computed by county.

#### Coverage

The KAIS 2007 and KENPHIA surveys covered the entire national territory, but KAIS 2012 excluded one geographic region, North Eastern. Therefore, to ensure that differences in coverage did not bias trend analyses, this region was omitted from the analysis, thereby stratifying by 17 remaining NASCOP region/residency strata across all three surveys.

#### Survey weighting

To compensate for over- or under- sampling of individuals or for disproportionate stratification along with the non-response, studies often include several types of survey weights in the datasets that are made available after the survey. Individual, child, and HIV-testing (blood) weights ensure that adults aged 15–64 years, children aged 0–14 years, and individuals selected for HIV testing, respectively, are representative of the population sampled. The survey design and nonresponse weighting approach for KAIS 2007 and KAIS 2012 were similar, and so no adjustments were made to the weights used in these studies. The KENPHIA 2018 survey design weights differed from the KAIS design weights in that no household-level post-stratification adjustments were done, and nonresponse weights were developed using a least absolute shrinkage and selection operator regression and chi-square automatic interaction detection methodology rather than the simpler inverse proportional weighting done by sex and geographic area variables. All the variables available in KENPHIA 2018, whether household, individual and blood draw specific, were used for this purpose [40]. Furthermore, post-stratification weights were developed to age and sex control totals from the national population projections for 2019 for KENPHIA. Therefore, to remove potential biases in comparisons resulting from the differing nonresponse and post-stratification weighting approaches, KENPHIA was reweighted to increase comparability between weighted estimates across the surveys.

#### Revised KENPHIA weights

A primary sampling unit (PSU) or enumeration area (EA) base weight was computed as the inverse of the probability of selection of the EA. No PSU nonresponse adjustment was made, apart from two ineligible EAs whose weights were set to 0. A household’s initial weight was then computed as a product of the PSU base weight and the inverse of the probability of selection of the household within the EA. An unknown eligibility household nonresponse adjustment was computed as a product of the household initial weight and the inverse of the probability of the household having unknown eligibility. The household weight was further adjusted for the eligible household member nonresponse rate.

Adult person-level weights were assumed equal to the household weight since all adults (aged ≥ 15 years) were eligible in a household. In the case of children (aged 0–14 years), only children in every third household were included in KENPHIA 2018. The child weight was then computed as three times the household weight. For adults, nonresponse adjustments cells were created by NASCOP region, urban–rural residence, and sex, whereas nonresponse-weighting classes for children were not stratified by sex. The post-stratification cells are produced by NASCOP region and sex. The child weights were not post stratified.

A similar approach was used to compute the HIV-testing (blood) weights included in the study.

### Data manipulation and merging

Using the three individual survey datasets, we created a dataset that included survey year, the design variables (weights, strata, and cluster), demographic characteristics, and HIV-specific indicators. The stratification variable in the combined dataset consisted of the 17 NASCOP regions. The cluster was uniquely characterized by the survey year and the cluster identifier in each survey. The weights in the combined dataset were normalized such that the normalized weights summed to the total number of respondents in each survey. The SAS program that combines the three datasets and renames and recodes variables to facilitate comparative analyses is available in Supplementary File 1.

To create the combined data file, we combined 2007, 2012, and 2018 files so that the number of respondents in the combined data file was the sum of the respondents from the three individual files. We then ensured that the analysis variables had the same names and values or categories in all three data files. Table 2 illustrates how the variables used in this analysis were redefined. Secondly, the approach to creating the new set of statistical weights is provided in Supplementary File 2.

**Table 2:** Variables included in the combined dataset for KAIS 2007, KAIS 2012 and KENPHIA 2018

Variable	Label	2007	2012	2018	Combined dataset
testedpastyear	Tested in the past year	Created by using the variables q612 and q612	Created by recoding the variable hivtest_year	**testedreceived12months**	1 = "Yes"; 0 = "No"
age	Age of respondent (5-year age ands)	Recoded the numeric variable Q103	Used the 10 category **agecat5**	Recoded the categorical variable **agegroup**	0 = “0–14”; 1 = “15–19”; 2 = “20–24”; 3 = “25–29”; 4 = “30–34”; 5 = “35–39”; 6 = “40–44”; 7 = “45–49”; 8 = “50–54”; 9 = “55–59”; 10 = “60–64”; 11 = “65 + ”;
sex	Sex of the respondent	Used the variable sex as provide in the dataset	Used the variable sex as provide in the dataset	Used the variable Sex as provide in the dataset	1 = “Male”; 2 = “Female”
residence	Place of residence	Recoded the categorical variable QRESID	Recoded the categorical variable qresid	Renamed the variable **urban**	1 = “Urban”; 2 = “Rural”
educ3	Level of education	Create from q104 and q105	Create from q103 and q104	Used the variable **SCHLAT**	1 = “No education”; 2 = “Primary”; 3 = “Secondary + ”
mstatus	Marital status	Recoded the categorical variable MARITAL1	Recoded the categorical variable marital3	Used the variables evermar and curmar create the marital status variable	1 = “Never married”; 2 = “Separated/Divorced/Widowed” 3 = “Married/Living together”
wealthind	Wealth index	Used the variable WLTHIND5	Used the variable windex5	Used the variable wealthquintile	1 = “Poorest”; 2 = "Poorer”; 3 = “Middle”; 4 = "Richer”; 5 = “Richest”
year	Year of survey	Created the variable, setting survey year to all respondents	Created the variable, setting survey year to all respondents	Created the variable, setting survey year to all respondents	1 = “2007”; 2 = “2012”; 3 = “2018”
agefirstsex	Age at first sex (in years)	Used the variable eversex and agefirstsex to create the age at first sex variable. The age at first sex for individuals who had not had sex by the date of the survey were rights censored and were treated as their age at that time	Used the variable eversex and agefirstsex to create the age at first sex variable. The age at first sex for individuals who had not had sex by the date of the survey were rights censored and were treated as their age at that time	Used the variable sexever and firstsxage to create the age at first sex variable. The age at first sex for individuals who had not had sex by the date of the survey were rights censored and were treated as their age at that time	Continuous variable
age1sex	Age at sexual debut	Similar to agefirstsex but used in the assessment of early sexual debut	Created in the same way as in the case of the 2007 survey	Created in the same way as in the case of the 2007 survey	Continuous variable
Cstatus	Censor status	A variable used in the time to event analysis which takes a value if the age1sex is less that 15 years and 0 otherwise	Created in the same way as in the case of the 2007 survey	Created in the same way as in the case of the 2007 survey	1 = “Experienced event”; 0 = “Censored”

*Abbreviations*: *KAIS* Kenya AIDS Indicator survey, *KENPHIA* Kenya Population-based HIV Impact Assessment

The study investigators did not interact with human subjects or have access to identifiable data or specimens. This was a secondary data analysis using anonymized data from each of the surveys that were included.

**Fig. 1:**
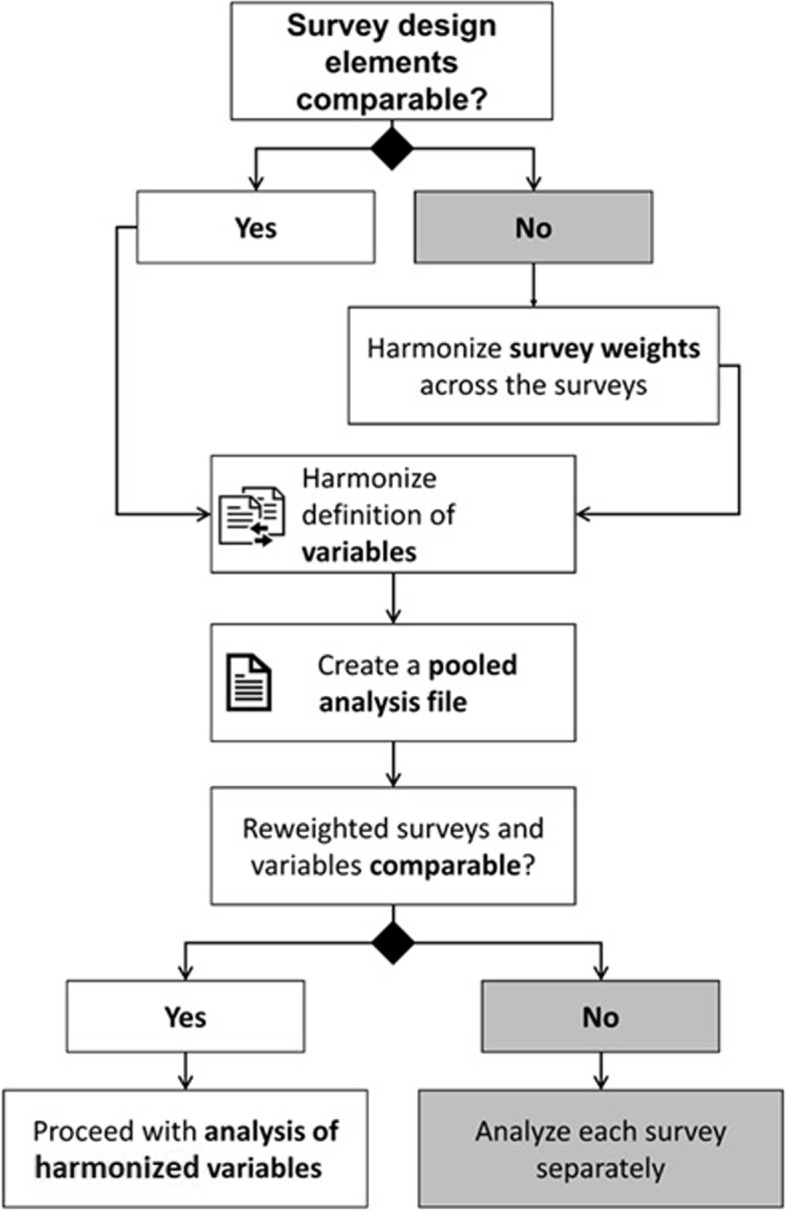
Preparing a trend analysis across independent surveys

Figure 1 describes our suggested approach for harmonization of variables and datasets to perform trend analysis.

### Assessing comparability of reweighted surveys across key population characteristics

Ideally, a set of unchanging population characteristics could be used to assess the comparability of the original and re-weighted datasets before proceeding with trend analyses. In the absence of such ideal variables, several demographic characteristics such as age, sex, marital status, residency, wealth index, and education, which have predictable trends and have been measured in other surveys over time, can be assessed for trends. In this analysis, we assessed the weighted distribution of each of these variables and used survey-weighted logistic regression to assess changes in the selected characteristics over time (Table 3). We found that there was no significant difference (trend) in key demographic variables selected for comparative assessment of original and re-weighted KENPHIA 2018 datasets.

**Table 3:** Comparison of the distribution of participants in the 2018 survey computed using the revised and the original KENPHIA weights

**Characteristic**	**Unweighted** **n**	**2018 with revised weights**		**2018 with KENPHIA original weights**	
		**Weighted %** **(or median)**	**95% CI** **(or IQR)**	**Weighted %** **(or median)**	**95% CI** **(or IQR)**
*Age at first sex*	27,825	16.9	(14.8—19.1)	16.8	(14.8—19.0)
*Tested in the last 12 months*					
No	10,028	44.4	(43.4—45.4)	43.9	(42.9—45.0)
Yes	12,764	55.6	(54.6—56.6)	56.1	(55.0—57.1)
**Total**	**22,792**	**100**		**100**	
*Sex*					
Male	12,374	49.3	(48.6—50.0)	49.5	(48.7—50.2)
Female	16,574	50.7	(50.0—51.4)	50.5	(49.8—51.3)
**Total**	**28,948**	**100**		**100**	
*Age*					
15–19	4951	17	(16.3—17.7)	18.9	(18.2—19.6)
20–24	4064	14.6	(13.8—15.4)	16.6	(15.8—17.4)
25–29	3841	13.7	(13.0—14.5)	14.9	(14.2—15.6)
30–34	3961	14	(13.4—14.5)	13.1	(12.6—13.6)
35–39	3006	10.4	(9.9—10.9)	10.6	(10.1—11.2)
40–44	2639	9	(8.6—9.5)	8	(7.6—8.5)
45–49	2073	6.8	(6.5—7.2)	6.4	(6.1—6.8)
50–54	1730	5.8	(5.4—6.1)	4.9	(4.6—5.1)
55–59	1438	4.8	(4.4—5.1)	3.7	(3.5—4.0)
60–64	1245	3.9	(3.6—4.1)	2.8	(2.6—3.0)
**Total**	**28,948**	**100**		**100**	
*Place of residence*					
Urban	10,752	39.3	(37.2—41.4)	37.9	(35.7—40.0)
Rural	18,196	60.7	(58.6—62.8)	62.1	(60.0—64.3)
**Total**	**28,948**	**100**		**100**	
*Level of education*					
No education	2309	6.4	(5.1—7.7)	5.7	(4.7—6.7)
Primary	14,613	47.4	(45.9—48.9)	47.4	(45.9—48.9)
Secondary +	12,024	46.2	(44.6—47.9)	46.9	(45.4—48.5)
**Total**	**28,946**	**100**		**100**	
*Marital status*					
Never married	8976	34.7	(33.7—35.6)	37.8	(36.8—38.8)
Separated/Divorced/Widowed	1888	6	(5.6—6.4)	5.2	(4.9—5.6)
Married/Living together	16,473	59.4	(58.3—60.4)	57	(55.9—58.1)
**Total**	**27,337**	**100**		**100**	
*Wealth index*					
Poorest	7058	19.3	(17.5—21.1)	19.5	(17.9—21.1)
Poorer	6574	21	(19.7—22.3)	21.8	(20.5—23.0)
Middle	6209	20.3	(19.1—21.5)	21	(19.7—22.2)
Richer	5370	19.7	(18.0—21.3)	19.5	(18.0—21.0)
Richest	3732	19.7	(17.5—22.0)	18.3	(16.2—20.4)
**Total**	**28,943**	**100**		**100**	

*Abbreviations*: *CI* Confidence Interval, *KENPHIA* Kenya Population-based HIV Impact Assessment

## Results

### Illustrative statistical analysis

**Fig. 2:**
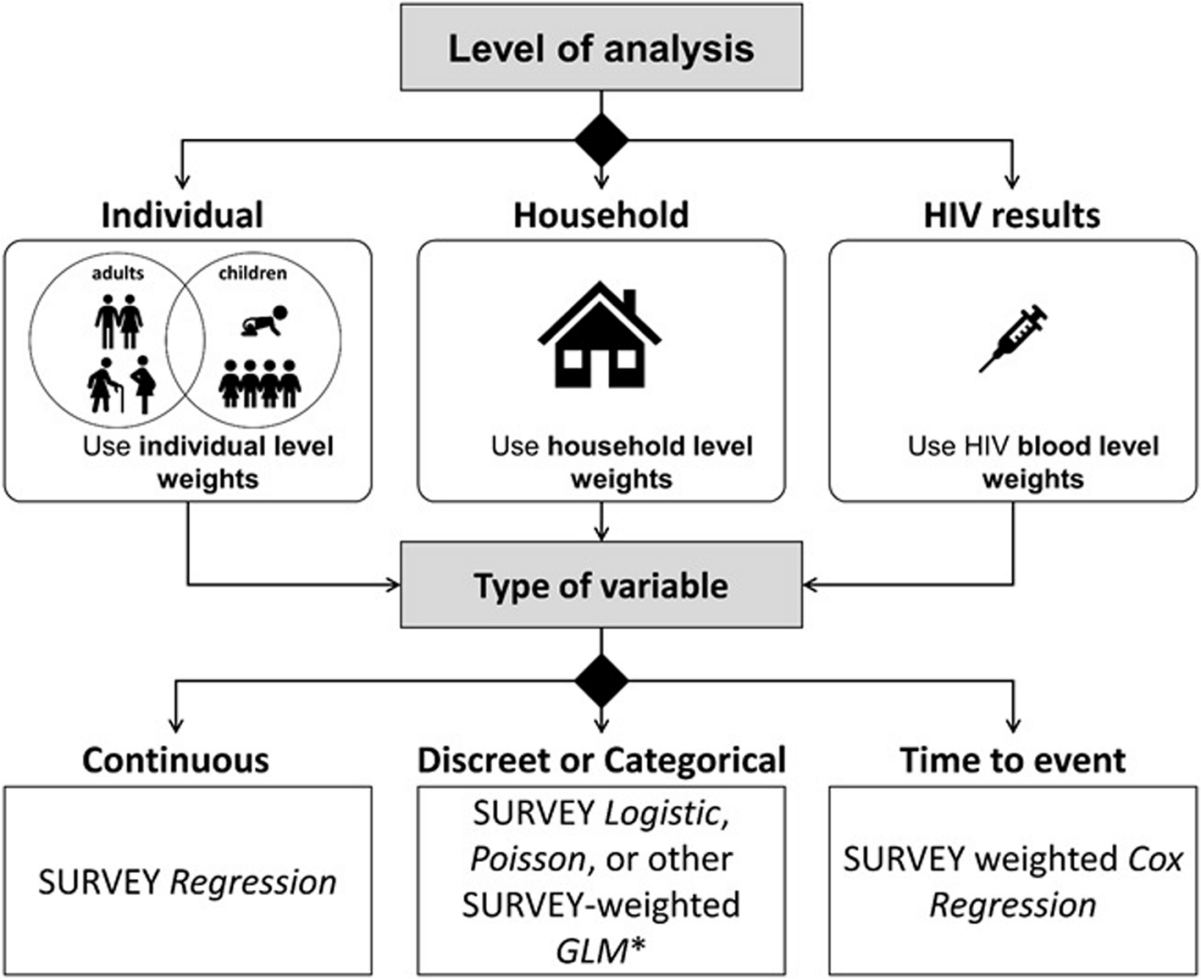
Choosing a statistical method based on type of variable to be analyzed in KAIS 2007, KAIS 2012 and KENPHIA 2018. Abbreviations: GLM, generalized linear model

Once the comparability of the revised and harmonized datasets is established, it is possible to carry out trend analysis on selected indicators. In our analysis, we selected trends in two behavioral indicators relevant to HIV programs: “Age of sexual debut among respondents aged 20–29 years” and “Tested for HIV in the past 12 months among respondents aged 15–64 years.” We selected these example indicators to illustrate trend analysis for continuous, binary, and time-to-event variables (Fig. 2). Trends were assessed visually and through regression methods, including adjustment for demographic variables to control for other changes in the population over time.

### Characteristics of the study population

Table 4 summarizes the sociodemographic characteristics of study participants. Women were overrepresented in all three surveys with male to female ratios of 1.00:1.33 in KAIS 2007, 1.00:1.38 in KAIS 2012, and 1.00:1.24 in KENPHIA 2018. There was a significant linear decline in the proportion of respondents sampled from within rural settings over time (KAIS 2007, 77.7% [95% confidence interval (CI): 75.1%–80.3%]; KAIS 2012, 62.9% [95% CI: 60.5%–65.3%]; and KENPHIA 2018, 60.7% [95% CI: 58.6%–62.8%]). There were significant variations in the distribution of the respondents by education. Across the three surveys, most respondents had primary education. Marital status varied between surveys. The age structure was generally consistent over time, except for a spike in the 20–24 year age group in 2007, followed by a similar spike in the 25–29 year age group in 2012 and in the 30–34 year age group in 2018. This pattern was consistent with an age cohort moving through the survey populations due to changing fertility or child mortality patterns in the mid-1980s.

**Table 4:** Demographic characteristics of interviewed study participants age 15–64 years in KAIS 2007, KAIS 2012 and KENPHIA 2018

	**2007**	**2012**	**2018**	**Total**	***P*-Value**
**Characteristic**	**Weighted % (95% CI)**	**Weighted % (95% CI)**	**Weighted % (95% CI)**	**Weighted % (95% CI)**	
Sex					< .001
Male	42.9(42.0—43.8)	49(48.0—50.0)	49.3(48.6—50.0)	46.8(46.1—47.6)	
Female	57.1(56.2—58.0)	51(50.0—52.0)	50.7(50.0—51.4)	53.2(52.4—53.9)	
Total	100	100	100	100	
Age of the respondent					< .001
15–19	16.8(16.0—17.5)	16.8(15.8—17.7)	17(16.3—17.7)	16.8(16.1—17.4)	
20–24	16.9(16.1—17.7)	16.3(15.4—17.1)	14.6(13.8—15.4)	16.5(15.9—17.1)	
25–29	13.9(13.3—14.6)	15.6(14.8—16.4)	13.7(13.0—14.5)	15(14.4—15.6)	
30–34	11.8(11.2—12.5)	12.3(11.7—13.0)	14(13.4—14.5)	12.2(11.7—12.6)	
35–39	10.5(9.9—11.0)	10.4(9.8—11.0)	10.4(9.9—10.9)	10.4(10.0—10.9)	
40–44	8.1(7.6—8.6)	8.5(8.0—9.0)	9(8.6—9.5)	8.4(8.0—8.8)	
45–49	7.9(7.4—8.4)	6.2(5.8—6.7)	6.8(6.5—7.2)	6.8(6.5—7.2)	
50–54	5.4(5.0—5.8)	6.1(5.7—6.6)	5.8(5.4—6.1)	5.9(5.5—6.2)	
55–59	5.2(4.8—5.6)	4.3(3.9—4.8)	4.8(4.4—5.1)	4.6(4.3—4.9)	
60–64	3.5(3.2—3.9)	3.4(3.0—3.8)	3.9(3.6—4.1)	3.5(3.2—3.7)	
Total	100	100	100	100	
Place of residence					< .001
Urban	22.3(19.7—24.9)	37.1(34.7—39.5)	39.3(37.2—41.4)	31.8(30.1—33.6)	
Rural	77.7(75.1—80.3)	62.9(60.5—65.3)	60.7(58.6—62.8)	68.2(66.4—69.9)	
Total	100	100	100	100	
Level of education					< .001
No education	10.6(9.5—11.7)	7.3(6.0—8.6)	6.4(5.1—7.7)	8.5(7.5—9.4)	
Primary	55.7(54.0—57.5)	59.4(57.3—61.5)	47.4(45.9—48.9)	58.1(56.6—59.6)	
Secondary +	33.7(31.8—35.5)	33.3(31.2—35.4)	46.2(44.6—47.9)	33.4(32.0—34.9)	
Total	100	100	100	100	
Marital status					< .001
Never married	29.2(28.1—30.3)	33.6(32.2—34.9)	34.7(33.7—35.6)	32(31.0—32.9)	
Separated/Divorced/Widowed	10.6(10.0—11.2)	4.2(3.8—4.6)	6(5.6—6.4)	6.5(6.1—6.9)	
Married/Living together	60.3(59.0—61.5)	62.3(60.9—63.7)	59.4(58.3—60.4)	61.5(60.6—62.5)	
Total	100	100	100	100	
Wealth index					< .001
Poorest	14.7(13.0—16.5)	19.2(16.6—21.8)	19.3(17.5—21.1)	17.6(15.8—19.4)	
Poorer	18.2(16.6—19.8)	20.8(18.9—22.7)	21(19.7—22.3)	19.9(18.5—21.2)	
Middle	20.3(18.8—21.8)	19.8(17.9—21.7)	20.3(19.1—21.5)	20(18.7—21.3)	
Richer	21.7(19.9—23.4)	19.3(17.2—21.4)	19.7(18.0—21.3)	20.1(18.6—21.6)	
Richest	25.1(22.4—27.8)	20.8(18.1—23.6)	19.7(17.5—22.0)	22.3(20.4—24.3)	
Total	100	100	100	100	

*Abbreviations*: *CI* Confidence Interval, *KAIS* Kenya AIDS Indicator survey, *KENPHIA* Kenya Population-based HIV Impact Assessment

Table 4 Demographic characteristics of interviewed study participants age 15–64 years in KAIS 2007, KAIS 2012 and KENPHIA 2018.

### Sexual debut

Trends in sexual debut were initially assessed visually and through regression methods, including adjusted analyses including demographic variables to control for other changes in the population over time using SAS PROC SURVEYREG. In this case, we assumed that the outcome was continuous and emanated from a Gaussian distribution. For this specific outcome variable, the analysis was restricted to individuals aged 20–29 years at the time of the survey. An Age-Period-Cohort (APC) analysis approach was used, with 2 age categories (20–24 years, 25–29 years), three time periods (2007, 2012 and 2018) and 5 birth-cohorts (1975–1979, 1980–1984, 1985–1989, 1990–1994 and 1995–1999).

Table 5 provides an example of how one can present summaries, trends, and regression results for the analysis of a continuous covariate such as age at sexual debut by selected covariates. In general, the median age at sexual debut of the study participants has increased significantly over time. There was a monotonic increase in the median age at sexual debut by age, level of education and wealth index. Age at sexual debut was consistently higher among older, and better educated individuals and individuals from the richest households. Age at sexual debut increased over time among the women, peaking in 2012 and decreasing slightly in 2018. Age at sexual debut was lower among the married respondents and those separated compared to those who never married. Age at sexual debut was lower among the married respondents and those separated, divorce or widowed compared to those who never married.

**Table 5:** Trends and regression results for age at first sex by selected covariates in KAIS 2007, KAIS 2012 and KENPHIA 2018

	**Summary Analysis**			**Survey weighted Regression**			
**Variable**	**N**	**Mean (95% CI)**	**Median (Range)**	**Unadjusted β (95% CI)**	***p*-value**	**Adjusted β (95% CI)**	***p*-value**
*Year of survey*							
2007	5093	17.45(17.32–17.58)	16.9(21)	1.00 (Reference)		1.00 (Reference)	
2012	4144	17.74(17.57–17.9)	17.2(28)	0.31(0.09–0.52)	0.0048	-0.24(-0.62–0.15)	0.2291
2018	8055	17.66(17.54–17.78)	17.2(45)	0.24(0.06–0.42)	0.009	-0.89(-1.68–0.11)	0.0251
*Sex*							
Female	10,504	17.77(17.63–17.91)	17.2(34)	1.00 (Reference)		1.00 (Reference)	
Male	6788	17.47(17.3–17.64)	16.9(45)	-0.33(-0.54–0.13)	0.0014	-0.96(-1.17–0.75)	< .0001
*Age*							
20–24	9097	17.44(17.32–17.56)	17.1(44)	1.00 (Reference)			
25–29	8195	17.86(17.69–18.03)	17.1(45)	0.38(0.2–0.56)	< 0.001	0.27(0.19–0.36)	< .0001
*Cohort*							
1975–1979	916	17.56(17.31–17.82)	16.9(21)	1.00 (Reference)		1.00 (Reference)	
1980–1984	3197	17.6(17.42–17.78)	16.9(28)	0.01(-0.29–0.31)	0.9525	0.56(0.14–0.97)	0.0086
1984–1989	4438	17.75(17.59–17.91)	17.2(28)	0.2(-0.09–0.5)	0.1811	1.25(0.53–1.96)	< 0.001
1990–1994	5366	17.47(17.27–17.66)	17.3(45)	-0.05(-0.38–0.28)	0.7678	1.56(0.56–2.57)	0.0024
1995–1999	3375	17.27(17.11–17.42)	17.1(27)	-0.27(-0.57–0.04)	0.0836	1.68(0.31–3.06)	0.0165
*Place of residence*							
Urban	7280	18.01(17.81–18.22)	17.4(45)	1.00 (Reference)		1.00 (Reference)	
Rural	10,012	17.38(17.24–17.51)	16.9(44)	-0.64(-0.9–0.39)	< 0.001	0.05(-0.23–0.34)	0.7261
*Level of education*							
No education	1307	16.31(15.79–16.84)	15.4(28)	1.00 (Reference)		1.00 (Reference)	
Primary	7807	17.09(16.96–17.22)	16.5(34)	0.8(0.27–1.34)	0.0032	0.66(0.17–1.15)	0.0085
Secondary +	8177	18.48(18.29–18.66)	17.9(45)	2.2(1.65–2.74)	< 0.001	1.68(1.16–2.19)	< .0001
*Marital status*							
Never married	6924	18.32(18.16–18.48)	17.8(44)	1.00 (Reference)		1.00 (Reference)	
Separated/Divorced/Widowed	539	16.65(16.3–17)	16(22)	-1.67(-2.04–1.29)	< 0.001	-1.78(-2.16–1.39)	< .0001
Married/Living together	9212	17.2(17.06–17.34)	16.6(45)	-1.12(-1.31–0.93)	< 0.001	-1.38(-1.58–1.18)	< .0001
*Wealth index*							
Poorest	3311	16.98(16.69–17.28)	16.4(44)	1.00 (Reference)		1.00 (Reference)	
Poorer	3011	17.2(16.96–17.45)	16.6(34)	0.23(-0.13–0.58)	0.2148	-0.01(-0.32–0.3)	0.9562
Middle	3114	17.34(17.13–17.54)	17(28)	0.38(0.02–0.74)	0.039	0.07(-0.25–0.4)	0.668
Richer	3621	17.75(17.54–17.95)	17.1(45)	0.79(0.43–1.15)	< 0.001	0.33(0–0.67)	0.0531
Richest	4235	18.31(18.08–18.55)	17.8(28)	1.33(0.95–1.71)	< 0.001	0.51(0.11–0.91)	0.0134

*Abbreviations*: *CI* Confidence Interval, *KAIS* Kenya AIDS Indicator survey, *KENPHIA* Kenya Population-based HIV Impact Assessment

In addition to assessing sexual debut as a continuous outcome variable, we also assessed trends in early sexual debut. Early sexual debut was defined as first vaginal intercourse before 15 years of age [19–22]. The time taken until first sexual intercourse for anyone who had not had sex by the age of 15 years was considered to be censored. We used the Kaplan–Meier method to compute the survival probability (not having become sexually active by age 15 years) by each age. We used SAS, version 9.4, to produce separate Kaplan–Meier estimates for each level of the covariates of interest. A log-rank test is not available for complex survey data to assess equality of survival curves, but Cox models are available for complex survey data. For our analyses, we used SAS PROC LIFETEST and SAS PROC SURVEYPHREG. The incidence of early sexual debut decreased, although not significantly, from 11.63(10.95–12.34) per 1,000 person-years at risk in 2007 to 10.45(9.75–11.2) per 1,000 person-years at risk in 2012 and further decreased significantly to 9.58(9.08–10.1) per 1,000 person-years at risk in 2018 (Table 6).

**Table 6:** Person-time analysis and survey-weighted Cox-regression of age at sexual debut by selected covariates in KAIS 2007, KAIS 2012 and KENPHIA 2018

Variable	Person-time analysis			Survey-weighted Cox-regression			
	**Event**	**Person-time**	**Rate (95% CI)**	**HR (95% CI)**	***p*-value**	**AHR (95% CI)**	***p*-value**
Year							
2007	1080	92,901	11.63(10.95–12.34)	1.00 (Reference)		1.00 (Reference)	
2012	793	75,892	10.45(9.75–11.2)	0.97(0.86–1.09)	0.5837	1.01(0.85–1.19)	0.9431
2018	1376	143,682	9.58(9.08–10.1)	0.8(0.71–0.89)	< 0.001	0.87(0.63–1.18)	0.3629
Cohort							
1975–1979	189	16,789	11.26(9.76–12.98)	1.00 (Reference)		1.00 (Reference)	
1980–1984	737	59,137	12.46(11.59–13.4)	1.22(0.99–1.5)	0.0574	1.27(1.01–1.59)	0.0408
1984–1989	824	80,902	10.19(9.51–10.9)	1.01(0.83–1.23)	0.8936	1.12(0.85–1.48)	0.4113
1990–1994	922	96,884	9.52(8.92–10.15)	0.88(0.69–1.11)	0.2693	1.07(0.73–1.55)	0.7431
1995–1999	577	58,763	9.82(9.05–10.65)	0.87(0.71–1.07)	0.1979	1.32(0.81–2.15)	0.2625
Age							
20–24	1638	161,535	10.14(9.66–10.64)	1.00 (Reference)		1.00 (Reference)	
25–29	1611	150,940	10.67(10.16–11.21)	1.17(1.05–1.31)	0.0047	0.96(0.81–1.13)	0.6292
Sex							
Male	1425	119,908	11.88(11.28–12.52)	1.00 (Reference)		1.00 (Reference)	
Female	1824	192,567	9.47(9.05–9.92)	1.05(0.92–1.2)	0.5055	0.76(0.67–0.86)	< .0001
Residence							
Urban	1170	133,603	8.76(8.27–9.27)	1.00 (Reference)		1.00 (Reference)	
Rural	2079	178,872	11.62(11.13–12.13)	1.31(1.13–1.5)	< 0.001	0.99(0.81–1.21)	0.8922
Education							
No education	472	23,989	19.68(17.98–21.53)	1.00 (Reference)		1.00 (Reference)	
Primary	1720	136,383	12.61(12.03–13.22)	0.45(0.37–0.54)	< 0.001	0.47(0.4–0.56)	< .0001
Secondary +	1057	152,085	6.95(6.54–7.38)	0.27(0.21–0.33)	< 0.001	0.32(0.26–0.4)	< .0001
Marital status							
Never married	977	127,821	7.64(7.18–8.14)	1.00 (Reference)		1.00 (Reference)	
Married/Living together	128	9459	13.53(11.38–16.09)	1.72(1.49–1.98)	< 0.001	1.57(1.37–1.8)	< .0001
Separated/Divorced/Widowed	2018	164,493	12.27(11.74–12.82)	1.94(1.5–2.49)	< 0.001	1.78(1.37–2.32)	< .0001
Wealth							
Poorest	908	58,409	15.55(14.57–16.59)	1.00 (Reference)		1.00 (Reference)	
Poorer	620	52,995	11.7(10.81–12.66)	0.76(0.62–0.94)	0.0115	0.91(0.76–1.08)	0.2772
Middle	598	55,856	10.71(9.88–11.6)	0.8(0.65–0.99)	0.0441	1.01(0.84–1.22)	0.919
Richer	566	65,946	8.58(7.9–9.32)	0.57(0.46–0.71)	< 0.001	0.75(0.61–0.93)	0.009
Richest	557	79,269	7.03(6.47–7.64)	0.55(0.44–0.68)	< 0.001	0.82(0.63–1.06)	0.1309

*Abbreviations*: *HR* Hazard rate, *AHR* Adjusted Hazard rate, *CI* Confidence Interval, *KAIS* Kenya AIDS Indicator survey, *KENPHIA* Kenya Population-based HIV Impact Assessment

### Tested in the last 12 months

We used SAS PROC SURVEYLOGISTIC to fit a survey-weighted logistic regression model to the binary outcome “Tested for HIV in the last 12 months.” Table 7 presents trends in the rates of HIV testing in the past 12 months among individuals aged 15–64 years. The results suggest a significant increase in the HIV-testing rates over time when adjusting for all the covariates considered. HIV-testing rates increased significantly from 12.6% (95% CI: 11.6%–13.6%) in 2007 to 56.1% (95% CI: 54.6%–57.6%) in 2012 but decreased slightly, although not significantly, to 55.6% [95% CI: 54.6%–56.6%) in 2018. Further, based on the survey-weighted logistic regression, after adjustment for all covariates considered, HIV testing rates increased substantially over time.

**Table 7:** Logistic regression results for HIV in the last 12 months by selected covariates in KAIS 2007, KAIS 2012 and KENPHIA 2018

Characteristic	Unweighted n/N	Prevalence (95% CI)	*p*-value	OR (95% CI)	*p*-value	Global *p*-value	AOR (95% CI)	*p*-value	Global *p*-value
Year of survey			< .001						
2007	1858/14392	12.6(11.6—13.6)		1.00 (Reference)			1.00 (Reference)		
2012	5526/9841	56.1(54.6—57.6)		8.87 (7.97–9.86)	< 0.001	< 0.001	8.45(7.59–9.42)	< 0.001	< 0.001
2018	12,764/22792	55.6(54.6—56.6)		8.69 (7.89–9.57)	< 0.001		8.62(7.81–9.51)	< 0.001	
Sex			< .001						
Female	12,782/27489	40.3(39.0—41.7)		1.00 (Reference)			1.00 (Reference)		
Male	7366/19536	37.2(35.6—38.8)		0.88 (0.82–0.94)	< 0.001	< 0.001	0.99(0.91–1.09)	0.9006	0.9006
Age of the respondent			< .001						
15–19	2384/6205	33.2(30.8—35.6)		1.00 (Reference)			1.00 (Reference)		
20–24	3858/7305	49.9(47.7—52.0)		2.00 (1.76–2.28)	< 0.001	< 0.001	1.4(1.21–1.62)	< 0.001	< 0.001
25–29	3584/7044	47.3(45.0—49.6)		1.81 (1.58–2.06)	< 0.001		1.05(0.91–1.22)	< 0.001	
30–34	3049/6574	41(38.7—43.3)		1.40 (1.22–1.61)	< 0.001		0.85(0.7–1.01)	0.3206	
35–39	2240/5278	38.2(35.8—40.6)		1.25 (1.08–1.43)	0.002		0.79(0.66–0.95)	0.9096	
40–44	1703/4388	34.5(31.9—37.1)		1.06 (0.91–1.24)	0.458		0.7(0.57–0.85)	0.0348	
45–49	1209/3574	27.1(24.4—29.7)		0.75 (0.63–0.88)	< 0.001		0.6(0.48–0.74)	< 0.001	
50–54	931/2818	31.1(27.9—34.3)		0.91 (0.77–1.07)	0.256		0.64(0.51–0.81)	0.0071	
55–59	677/2197	27(23.5—30.5)		0.75 (0.61–0.91)	0.004		0.68(0.51–0.9)	0.1008	
60–64	513/1642	25(20.5—29.5)		0.67 (0.52–0.87)	0.002		0.59(0.43–0.8)	0.0132	
Place of residence			< .001						
Urban	8309/16687	49.2(47.1—51.3)		1.00 (Reference)			1.00 (Reference)		
Rural	11,839/30338	33.7(32.2—35.2)		0.53 (0.47–0.59)	< 0.0011	< 0.001	0.89(0.77–1.01)	0.078	0.078
Level of education			< 0.001						
No education	1386/4096	27.4(24.0—30.8)		1.00 (Reference)			1.00 (Reference)		
Primary	9961/24441	38(36.6—39.5)		1.63 (1.36–1.95)	< 0.001	< 0.001	1.04(0.88–1.23)	0.4343	0.0288
Secondary +	8801/18487	42.7(40.9—44.5)		1.97 (1.64–2.37)	< 0.001		1.17(0.98–1.4)	0.0204	
Marital status			< .001						
Never married	5441/12943	38(36.1—39.8)		1.00 (Reference)			1.00 (Reference)		
Married/Living together	1185/3505	24.6(22.0—27.3)		1.09 (1.00–1.19)	0.05	< 0.001	1.23(1.09–1.39)	0.0396	0.005
Separated/Divorced/Widowed	12,470/28661	40(38.6—41.4)		0.53 (0.46–0.62)	< 0.001		1.21(0.98–1.49)	0.3686	
Wealth index			< .001						
Poorest	3876/8791	35.9(33.0—38.8)		1.00 (Reference)			1.00 (Reference)		
Middle	4138/9825	37.4(34.9—39.9)		1.07 (0.90–1.26)	0.451	< 0.001	1.05(0.9–1.21)	0.0597	0.0307
Poorer	3996/9613	36.2(33.6—38.8)		1.01 (0.87–1.18)	0.866		1.15(0.99–1.34)	0.8571	
Richer	4095/9457	39.5(37.1—41.9)		1.16 (0.98–1.37)	0.076		1.2(1.02–1.4)	0.2948	
Richest	4042/9337	43.6(41.2—46.1)		1.38 (1.17–1.63)	< 0.001		1.36(1.11–1.67)	0.0059	

*Abbreviations***:** *OR*-Odds ratio, *AOR* Adjusted Odds ratio, *CI* Confidence Interval, *KAIS* Kenya AIDS Indicator survey, *KENPHIA* Kenya Population-based HIV Impact Assessment

In Fig. 2, we provide a rubric that can be used to make decisions about the statistical analysis to employ for a given analysis question based on various design considerations. The SAS program used to carry out the crosstabulation, the survey-weighted regression analysis, the survey-weighted logistic regression analysis, the survey-weighted regression, and the person-time analysis is available in Supplementary File 3.

## Discussion

We developed an approach for assessing and harmonizing independent population-based surveys to assess trends in HIV-related indicators. After describing the methods used to design and weight each survey, we harmonized stratification, demographic variables, and survey weights to ensure comparability before proceeding with a trend analysis. In this analysis, the survey weights for the latest survey (KENPHIA 2018) were revised to ensure comparability with the previous two surveys. It is important to note that we developed these methods strictly to allow for comparisons between surveys. The methods are not meant to provide revised or improved estimates for the most recent survey analyzed (KENPHIA). The original weights for KENPHIA are optimal and should be used to analyze and present the results of the KENPHIA survey. Similar approaches to making comparisons between surveys are documented elsewhere in the literature [23–30]. For reproducibility, we also provide the analysis codes that demonstrate how the analysis was carried out and how the comparison was done.

The weighted distributions of demographic variables were consistent across surveys with some exceptions. There was an increasing proportion of the sample that resided in urban areas, as expected given broad development trends in Kenya. The age structure showed spikes in subsequent age groups across surveys, consistent with a cohort effect from reductions in fertility 15–20 years before the KAIS 2007 survey, given that the surveys were spaced at approximately 5-year intervals, consistent with the historical fertility reductions observed in the recent Demographic and Health survey [31] and census [13] in Kenya. Other differences are difficult to explain. For example, the sex distribution seemed skewed in 2007, with 42% of the survey population being men, compared to higher proportions of in the other two surveys (48%–49%), perhaps indicating coverage issues among men in that survey.

We used two outcome variables expected to change over time (HIV testing and age at sexual debut) to demonstrate a methodology to carry out trend comparisons. For HIV testing in the last 12 months, we highlighted two approaches that can be used to assess trends in dichotomous outcomes. We first computed survey-weighted proportions and plotted the resulting trends over time by selected covariates. We then fitted logistic regression models with the year as a covariate, adjusting for age, sex, residence, marital status, and wealth index. This approach has been employed in several other previous studies. Trends in HIV-testing rates have also been discussed extensively in the literature [23–25]. The use of chi-square tests of trends and logistic regression were extensively used in the literature.

For age at sexual debut, we show two approaches for assessing trends for a continuous variable. Several studies have also used survey data from low-income countries to assess trends in the HIV-related outcomes considered in our analysis. Several studies have treated age at sexual debut as a time-to-event outcome, assessing this outcome variable's trends among different cohorts observed [32, 33]. These studies have used survival analysis-based approaches to assess trends in the outcome variables of interest. In our analysis, we used two approaches where the first ignored censoring in the age at sexual debut and presented a summary and regression-based results as an example of how trends in continuous outcome variables could be assessed [34]. We then used the survival approach and found a decrease in the risk of early sexual debut over time.

Our analysis is subject to several limitations. The trend comparison was based on using three time points (2007, 2012, and 2018), so we were only able to make relatively short-term assessments of the trajectory of the indicators considered. Previous studies used Cochran Armitage chi-square tests or Z-tests to assess the significance of trends [35–38]. Survey weighted versions of this statistic were not implemented in our analysis due to limitations in the software we used. Another challenge encountered was the change in the definition of certain variables and indicators over time, adding uncertainty in interpreting the meaning of observed trends. Furthermore, not all questions asked across the three surveys were the same, making it difficult to analyze some of the outcomes across the three surveys. Our study did not address all relevant issues for every conceivable trend analysis that could be conducted with these surveys. For example, changes in HIV-testing algorithms may affect estimates. Great care is needed in interpreting results with potential underlying methodological differences. Finally, there are alternatives to population-based surveys for measuring trends in health conditions. For example, in 2018, Kenya established an HIV Case-Based Surveillance system to measure progress along the HIV care cascade to provide high-quality, timely, and reliable HIV data by population characteristics. Despite current limitations and challenges, this system will provide an opportunity for future assessment of trends based on a census of events rather than population-based sampling, as presented here.

The pooling of data from multi-year surveys aimed at assessing trends in key public health performance indicators is an important part of interrogating the impact of programs and interventions. However, this pooling of data from large complex surveys leads to data sets with large sample sizes which inadvertently increase statistical power [39]. This increased power leads to a tendency of finding statistically significant differences however small they are. Therefore, researchers need to distinguish between statistical difference and scientific difference. The statistically significant difference that arises from larger sample sizes may not be scientifically meaningful.

## Conclusion

We have provided approaches and considerations that can be used to support trend comparisons for various outcome variables in HIV surveys in low-income settings. Our approach has demonstrated that independent national household surveys conducted over time can be assessed for comparability, adjusted as appropriate, and used to estimate trends in key indicators. Analyzing trends over time can not only provide insights into Kenya’s progress toward HIV epidemic control but also identify gaps in key HIV indicators.

## Supplementary Information

**Additional file 1: Supplementary file 1.** SAS Code that merged from three National HIV Population-based Household surveys in Kenya (2007, 2012, and 2018).

**Additional file 2: Supplementary File 2.** SAS code for the analysis of the outcome variable Age at first sex.

**Additional file 3: Supplementary File 3.** Proposal for the reweighting of variable in the 2018 Kenya Population-based HIV Impact Assessment (KENPHIA) survey data to match the 2012 Kenya AIDS indicator survey approach.

## Data Availability

The datasets used and/or analyzed during the current study are available from the corresponding author upon reasonable request.

## Abbreviations

KAIS: Kenya AIDS Indicator surveys
KENPHIA: Kenya Population-based HIV Impact Assessment
ART: Antiretroviral therapy
SSA: Sub-Saharan Africa
NHANES: National Health and Nutrition Examination Survey
NASSEP: National Sample Survey and Evaluation Programme
KNBS: Kenya National Bureau of Statistics
NASCOP: National AIDS and STI Control Programme
PSU: Primary sampling unit
EA: Enumeration area
KEMRI: Kenya Medical Research Institute
IRB: Institutional Review Board
CDC: Centers for Disease Control and Prevention
UCSF: University of California, San Francisco
CI: Confidence Interval
OR: Odds ratio
AOR: Odds ratio
DHS: Demographic and Health survey
