# Supplementary material for: Methods for conducting trends analysis: roadmap for comparing outcomes from three national HIV Population-based household surveys in Kenya (2007, 2012, and 2018)
Source: BMC Public Health. 2022 Jul 13;22:1337. doi: 10.1186/s12889-022-13633-8 (PMC9281165; doi:10.1186/s12889-022-13633-8)
Supplement: Supplementary file 1 — Additional file 1: Supplementary file 1. SAS Code that merged from three National HIV Population-based Household surveys in Kenya (2007, 2012, and 2018). [file 12889_2022_13633_MOESM1_ESM.docx]

Supplementary file 1. SAS Code that merged from three National HIV Population-based Household surveys in Kenya (2007, 2012, and 2018)

*-----------------------------------------------------------------------------------------------------------------------------;

* COMBINED DATASET;

* Purpose: This code combines the 3 survey datasets and creates a datafile combined.sas7bdat that is used

* in the subsequent analysis

* Date created: September 15, 2020

* Last Modified: April 8, 2021

* Author: Thomas Achia

* Dependencies: This file uses the SAS datasets datasets.kais2007 datasets.kais2012 datasets.kenphia2018

* stored in the directory C:\trends\datasets

* The resulting dataset is stored in the folder C:\trends\datasets

*-----------------------------------------------------------------------------------------------------------------------------;

dm 'odsresults; clear; log; clear; out; clear';

* to remove all datasets from within the WORK folder;

**proc** **datasets** lib=work nolist kill; **quit**; **run**;

* set working directory;

%let dir=C:\trends\;

* set output directory;

%let outdir=&dir.\output\tables;

* Load the formats to be used for both KENPHIA and KAIS;

%include "&dir.\data\myfmts.sas";

LIBNAME formats "&dir.\formats";

OPTIONS FMTSEARCH=(formats);

* Import the cleaned KAIS 2012 and KENPHIA 2018 data sets;

*%include "&dir.\codes\cleankenphia2018.sas";

*%include "&dir.\codes\cleankais2007.sas";

*%include "&dir.\codes\cleankais2012.sas";

* Specify the directory where the dataset will be stored";

libname datasets 'C:\trends\data';

* Work from the WORK library;

**data** kais2007;

set datasets.kais2007;

**run**;

**data** kais2012;

set datasets.kais2012;

**run**;

**data** datasets.combined;

set datasets.kais2007 datasets.kais2012 datasets.kenphia2018;

**run**;
