# Supplementary material for: Methods for conducting trends analysis: roadmap for comparing outcomes from three national HIV Population-based household surveys in Kenya (2007, 2012, and 2018)
Source: BMC Public Health. 2022 Jul 13;22:1337. doi: 10.1186/s12889-022-13633-8 (PMC9281165; doi:10.1186/s12889-022-13633-8)
Supplement: Supplementary file 2 — Additional file 2: Supplementary File 2. SAS code for the analysis of the outcome variable Age at first sex. [file 12889_2022_13633_MOESM2_ESM.docx]

Supplementary File 2. SAS code for the analysis of the outcome variable Age at first sex

*-----------------------------------------------------------------------;

* SURVEY REGRESSION TREND ANALYSIS FOR AGE AT FIRST SEX; *

* Purpose: This code provides the analysis of the outcome variable Age at first sex used to illustrate a

* analysis for a continuous outcome variable as part of the manuscript: "Methods for conducting trends * analysis: Roadmap for comparing outcomes from National Population-based Household surveys in

* Kenya, 2007, 2012, and 2018

*

* Date created: September 15, 2020

* Last Modified: April 8, 2021

* Author: Thomas Achia

* Dependencies: This file uses:

* 1. the file combined.sas7bdat

* 2. The SAS macros: svy_freqs_jrr_dev_09_25_20.sas and

* svy_logistic_regression.sas

* 3. The formats file: myfmts.sas

*-----------------------------------------------------------------------;

dm 'odsresults; clear; log; clear; out; clear';

* to remove kenphia datasets from within the WORK folder;

**proc** **datasets** lib=work nolist kill; **quit**; **run**;

* set working directory;

%let dir=C:\trends\;

* set output directory;

%let outdir=&dir.\output\tables;

libname datasets "&dir.\data";

* set output directory;

%let outdir=&dir.\output\tables;

* load required macros;

%include "&dir.\macros\svy_freqs_jrr_dev_09_25_20.sas";

%include "&dir.\macros\svy_logistic_regression.sas";

*Include the FORMATS file;

%include 'C:\trends\data\myfmts.sas';

* data steps ...;

**data** combined;

set datasets.combined;

if age>=**1**;

freq=**1**;

domain_kenphia=**1**;

age15_49=**0**;

if **1** <= age <=**7** then age15_49=**1**;

age15_64=**0**;

if **1** <= age <=**10** then age15_64=**1**;

age25_64=**0**;

if **3** <= age <=**10** then age25_64=**1**;

neprov=**1**;

if qprov=**5** then neprov=**0**;

* Indicator for all provinces minus NE prov;

age15_49minusne=neprov*age15_49;

age15_64minusne=neprov*age15_64;

age25_64minusne=neprov*age25_64;

* replace missing values code with .;

*array a(*) _numeric_;

*do i=1 to dim(a);

*if a(i) = . then a(i) = 99;

*end;

*drop i;

**run**;

**data** combined1;

set combined;

* if age<8;

hivtestever1=hivtestever;

if hivtestever=**99** then hivtestever1=**.**;

* Early sexual debut;

age1sex=**.**;

if agefirstsex>=**8** then age1sex= agefirstsex;

if agefirstsex<**8** then age1sex=**8**;

cstatus=**.**;

if age1sex<**15** then cstatus=**1**;

if age1sex>=**15** then cstatus=**0**;

agefirstsex1=agefirstsex;

if agefirstsex=**99** then agefirstsex1=**.**;

mstatus1=mstatus;

if mstatus=**99** then mstatus1=**.**;

age1=age;

if age=**0** then age1=**.**;

format age1 age1Fmt.;

**run**;

*---------------------------------------------------;

* This section computes the mean and median ages at

* first sex;

*---------------------------------------------------;

* The Mean;

**proc** **surveymeans** data=combined1;

weight intwt; strata strata; cluster qclust;

var agefirstsex1 ; domain age25_64minusne*year age25_64minusne*sex age25_64minusne*age age25_64minusne*residence age25_64minusne*educ3 age25_64minusne*mstatus age25_64minusne*wealthind;

* ods output domain =age25_64minusne ;

*format educ1 educFmt.;

**run** ;

* The Median;

**proc** **surveymeans** data=combined1 median;

weight intwt; strata strata; cluster qclust;

var agefirstsex1 ; domain year sex age residence educ3 mstatus wealthind;

ods output domain =age25_64minusne ;

*format educ1 educFmt.;

**run** ;

*---------------------------------------------------;

* This carries out simple linear regression analysis

for the primary outcome (year) and other covariates;

*---------------------------------------------------;

* Year;

**proc** **surveyreg** data=combined1 order=formatted;

class year (ref='2007') sex (ref = 'Female') age (ref = '25-29') residence (ref = 'Urban') educ3 (ref = 'No education') mstatus (ref = 'Never married') wealthind (ref = 'Poorest');

model agefirstsex1 = year/ solution clparm;

weight intwt; strata strata; cluster qclust;

domain age25_64minusne;

**run**;

* Sex;

**proc** **surveyreg** data=combined1 order=formatted;

class year (ref='2007') sex (ref = 'Female') age (ref = '25-29') residence (ref = 'Urban') educ3 (ref = 'No education') mstatus (ref = 'Never married') wealthind (ref = 'Poorest');

model agefirstsex1 = sex/ solution clparm;

weight intwt; strata strata; cluster qclust;

domain age25_64minusne;

**run**;

* Age;

**proc** **surveyreg** data=combined1 order=formatted;

class year (ref='2007') sex (ref = 'Female') age (ref = '25-29') residence (ref = 'Urban') educ3 (ref = 'No education') mstatus (ref = 'Never married') wealthind (ref = 'Poorest');

model agefirstsex1 = age/ solution clparm;

weight intwt; strata strata; cluster qclust;

domain age25_64minusne;

**run**;

* Residence;

**proc** **surveyreg** data=combined1 order=formatted;

class year (ref='2007') sex (ref = 'Female') age (ref = '25-29') residence (ref = 'Urban') educ3 (ref = 'No education') mstatus (ref = 'Never married') wealthind (ref = 'Poorest');

model agefirstsex1 = residence/ solution clparm;

weight intwt; strata strata; cluster qclust;

domain age25_64minusne;

**run**;

* Education;

**proc** **surveyreg** data=combined1 order=formatted;

class year (ref='2007') sex (ref = 'Female') age (ref = '25-29') residence (ref = 'Urban') educ3 (ref = 'No education') mstatus (ref = 'Never married') wealthind (ref = 'Poorest');

model agefirstsex1 = educ3/ solution clparm;

weight intwt; strata strata; cluster qclust;

domain age25_64minusne;

**run**;

* Marital status;

**proc** **surveyreg** data=combined1 order=formatted;

class year (ref='2007') sex (ref = 'Female') age (ref = '25-29') residence (ref = 'Urban') educ3 (ref = 'No education') mstatus (ref = 'Never married') wealthind (ref = 'Poorest');

model agefirstsex1 = mstatus/ solution clparm;

weight intwt; strata strata; cluster qclust;

domain age25_64minusne;

**run**;

* Wealth index;

**proc** **surveyreg** data=combined1 order=formatted;

class year (ref='2007') sex (ref = 'Female') age (ref = '25-29') residence (ref = 'Urban') educ3 (ref = 'No education') mstatus (ref = 'Never married') wealthind (ref = 'Poorest');

model agefirstsex1 = wealthind/ solution clparm;

weight intwt; strata strata; cluster qclust;

domain age25_64minusne;

**run**;

* Multivariable Analysis;

*---------------------------------------------------;

* This carries out multivariable linear regression

analysis

for the primary outcome (year) and other covariates;

*---------------------------------------------------;

**proc** **surveyreg** data=combined1;

cluster qclust;

class year (ref='2007') sex (ref = 'Female') age (ref = '25-29') residence (ref = 'Urban') educ3 (ref = 'No education') mstatus (ref = 'Never married') wealthind (ref='Poorest');

model agefirstsex1 = year sex age residence educ3 mstatus wealthind/ solution clparm;

weight intwt; strata strata; cluster qclust;

domain age25_64minusne;

**run**;

* The Mean;

**proc** **surveymeans** data=combined1;

weight intwt; strata strata; cluster qclust;

var agefirstsex1 ; domain age25_64minusne*year age25_64minusne*sex age25_64minusne*age age25_64minusne*residence age25_64minusne*educ3 age25_64minusne*mstatus age25_64minusne*wealthind;

* ods output domain =age25_64minusne ;

*format educ1 educFmt.;

**run** ;

* The Median;

**proc** **surveymeans** data=combined1 median;

weight intwt; strata strata; cluster qclust;

var agefirstsex1 ; domain age25_64minusne*year age25_64minusne*sex age25_64minusne*age age25_64minusne*residence age25_64minusne*educ3 age25_64minusne*mstatus age25_64minusne*wealthind;

*ods output domain =age25_64minusne ;

*format educ1 educFmt.;

**run** ;

*-----------------------------------------------------------------------;

* SURVEY REGRESSION TREND ANALYSIS FOR EARLY SEXUAL DEBUT; *

* Purpose: This code provides the analysis of the outcome *

* variable "Early sexual debut" used to illustrate a *

* trendanalysis for a time-to-evemt outcome variable *

* Part of the manuscript: "Methods for conducting trends*

* analysis: Roadmap for comparing outcomes from 3 *

* National Population-based Household surveys in Kenya, *

* 2007, 2012, and 2018" submitted to BMC public health: *

* Biostatistics and Methods

*

* Date created: September 15, 2020

* Last Modified: April 8, 2021

* Author: Thomas Achia

* Dependencies: This file uses:

* 1. the file combined.sas7bdat

* 2. The SAS macros: svy_freqs_jrr_dev_09_25_20.sas and

* svy_logistic_regression.sas

* 3. The formats file: myfmts.sas

*-----------------------------------------------------------------------;

dm 'odsresults; clear; log; clear; out; clear';

* to remove kenphia datasets from within the WORK folder;

**proc** **datasets** lib=work nolist kill; **quit**; **run**;

* set working directory;

%let dir=C:\trends\;

* set output directory;

%let outdir=&dir.\output\tables;

libname datasets "&dir.\data";

* set output directory;

%let outdir=&dir.\output\tables;

* load required macros;

%include "&dir.\macros\svy_freqs_jrr_dev_09_25_20.sas";

%include "&dir.\macros\svy_logistic_regression.sas";

*Include the FORMATS file;

%include 'C:\trends\data\myfmts.sas';

* data steps ...;

* data steps ...;

**data** combined;

set datasets.combined;

if age>=**1**;

freq=**1**;

domain_kenphia=**1**;

age15_49=**0**;

if **1** <= age <=**7** then age15_49=**1**;

age15_64=**0**;

if **1** <= age <=**10** then age15_64=**1**;

age25_64=**0**;

if **3** <= age <=**10** then age25_64=**1**;

neprov=**1**;

if qprov=**5** then neprov=**0**;

* Indicator for all provinces minus NE prov;

age15_49minusne=neprov*age15_49;

age15_64minusne=neprov*age15_64;

age25_64minusne=neprov*age25_64;

* replace missing values code with .;

*array a(*) _numeric_;

*do i=1 to dim(a);

*if a(i) = . then a(i) = 99;

*end;

*drop i;

**run**;

**data** combined1;

set combined;

* if age<8;

hivtestever1=hivtestever;

if hivtestever=**99** then hivtestever1=**.**;

* Early sexual debut;

age1sex=**.**;

if agefirstsex>=**8** then age1sex= agefirstsex;

if agefirstsex<**8** then age1sex=**8**;

cstatus=**.**;

if age1sex<**15** then cstatus=**1**;

if age1sex>=**15** then cstatus=**0**;

agefirstsex1=agefirstsex;

if agefirstsex=**99** then agefirstsex1=**.**;

mstatus1=mstatus;

if mstatus=**99** then mstatus1=**.**;

age1=age;

if age=**0** then age1=**.**;

format age1 age1Fmt.;

**run**;

* Kaplan-Meier survival curves;

**proc** **freq** data=combined1;

tables age year;

**run**;

**proc** **lifetest** data=combined1 atrisk plots=survival(atrisk cb) outs=outwhas500;

strata year;

time age1sex*cstatus(**0**);

**run**;

*Computing median survival time;

**proc** **lifetest** data=combined1 alphaqt=**0.05**;

time age1sex*cstatus(**0**);

strata year;

**run**;

**proc** **lifetest** data=combined1 atrisk plots=survival(atrisk cb) outs=outwhas500;

strata sex;

time age1sex*cstatus(**0**);

**run**;

**proc** **lifetest** data=combined1 atrisk plots=survival(atrisk cb) outs=outwhas500;

strata age;

time age1sex*cstatus(**0**);

**run**;

**proc** **lifetest** data=combined1 atrisk plots=survival(atrisk cb) outs=outwhas500;

strata residence;

time age1sex*cstatus(**0**);

**run**;

**proc** **lifetest** data=combined1 atrisk plots=survival(atrisk cb) outs=outwhas500;

strata educnew;

time age1sex*cstatus(**0**);

**run**;

**proc** **lifetest** data=combined1 atrisk plots=survival(atrisk cb) outs=outwhas500;

strata mstatus;

time age1sex*cstatus(**0**);

**run**;

**proc** **lifetest** data=combined1 atrisk plots=survival(atrisk cb) outs=outwhas500;

strata wealthind;

time age1sex*cstatus(**0**);

**run**;

* Person-time analysis;

* Rates by year;

**proc** **summary** data=combined1 nway;

var cstatus age1sex;

class year;

output out=rates(drop=_type_ _freq_) sum=cstatus age1sex;

**run**;

**data** rates;

set rates;

_rate=**1000***(eversexstatus/agefirstsex1);

ci_low=_rate/exp(**1.96***sqrt(**1**/eversexstatus));

ci_high=_rate*exp(**1.96***sqrt(**1**/eversexstatus));

**run**;

**proc** **print** noobs;

title ’Table of cases, person-years, and rates per **1000** person-years’;

var year cstatus age1sex _rate ci_low ci_high;

format agefirstsex1 **8.1** _rate ci_low ci_high **7.2**;

**run**;

* Rates by sex;

**proc** **summary** data=combined1 nway;

var cstatus age1sex;

class sex;

output out=rates(drop=_type_ _freq_) sum=cstatus age1sex;

**run**;

**data** rates;

set rates;

_rate=**1000***(eversexstatus/agefirstsex1);

ci_low=_rate/exp(**1.96***sqrt(**1**/eversexstatus));

ci_high=_rate*exp(**1.96***sqrt(**1**/eversexstatus));

**run**;

**proc** **print** noobs;

title ’Table of cases, person-years, and rates per **1000** person-years’;

var sex cstatus age1sex _rate ci_low ci_high;

format agefirstsex1 **8.1** _rate ci_low ci_high **7.2**;

**run**;

* Rates by Age;

**proc** **summary** data=combined1 nway;

var cstatus age1sex;

class age;

output out=rates(drop=_type_ _freq_) sum=cstatus age1sex;

**run**;

**data** rates;

set rates;

_rate=**1000***(eversexstatus/agefirstsex1);

ci_low=_rate/exp(**1.96***sqrt(**1**/eversexstatus));

ci_high=_rate*exp(**1.96***sqrt(**1**/eversexstatus));

**run**;

**proc** **print** noobs;

title ’Table of cases, person-years, and rates per **1000** person-years’;

var age cstatus age1sex _rate ci_low ci_high;

format agefirstsex1 **8.1** _rate ci_low ci_high **7.2**;

**run**;

* Rates by Residence;

**proc** **summary** data=combined1 nway;

var cstatus age1sex;

class residence;

output out=rates(drop=_type_ _freq_) sum=cstatus age1sex;

**run**;

**data** rates;

set rates;

_rate=**1000***(eversexstatus/agefirstsex1);

ci_low=_rate/exp(**1.96***sqrt(**1**/eversexstatus));

ci_high=_rate*exp(**1.96***sqrt(**1**/eversexstatus));

**run**;

**proc** **print** noobs;

title ’Table of cases, person-years, and rates per **1000** person-years’;

var residence cstatus age1sex _rate ci_low ci_high;

format agefirstsex1 **8.1** _rate ci_low ci_high **7.2**;

**run**;

* Rates by Education;

**proc** **summary** data=combined1 nway;

var cstatus age1sex;

class educnew;

output out=rates(drop=_type_ _freq_) sum=cstatus age1sex;

**run**;

**data** rates;

set rates;

_rate=**1000***(eversexstatus/agefirstsex1);

ci_low=_rate/exp(**1.96***sqrt(**1**/eversexstatus));

ci_high=_rate*exp(**1.96***sqrt(**1**/eversexstatus));

**run**;

**proc** **print** noobs;

title ’Table of cases, person-years, and rates per **1000** person-years’;

var educnew cstatus age1sex _rate ci_low ci_high;

format agefirstsex1 **8.1** _rate ci_low ci_high **7.2**;

**run**;

* Rates by Marital statu;

**proc** **summary** data=combined1 nway;

var cstatus age1sex;

class mstatus;

output out=rates(drop=_type_ _freq_) sum=cstatus age1sex;

**run**;

**data** rates;

set rates;

_rate=**1000***(eversexstatus/agefirstsex1);

ci_low=_rate/exp(**1.96***sqrt(**1**/eversexstatus));

ci_high=_rate*exp(**1.96***sqrt(**1**/eversexstatus));

**run**;

**proc** **print** noobs;

title ’Table of cases, person-years, and rates per **1000** person-years’;

var mstatus cstatus age1sex _rate ci_low ci_high;

format agefirstsex1 **8.1** _rate ci_low ci_high **7.2**;

**run**;

* Rates by Wealth index;

**proc** **summary** data=combined1 nway;

var cstatus age1sex;

class wealthind;

output out=rates(drop=_type_ _freq_) sum=cstatus age1sex;

**run**;

**data** rates;

set rates;

_rate=**1000***(eversexstatus/agefirstsex1);

ci_low=_rate/exp(**1.96***sqrt(**1**/eversexstatus));

ci_high=_rate*exp(**1.96***sqrt(**1**/eversexstatus));

**run**;

**proc** **print** noobs;

title ’Table of cases, person-years, and rates per **1000** person-years’;

var wealthind cstatus age1sex _rate ci_low ci_high;

format agefirstsex1 **8.1** _rate ci_low ci_high **7.2**;

**run**;

* Survival analysis to assess trend;

* The Unadjusted results;

**proc** **surveyphreg** data=combined1;

weight intwt; strata strata; cluster qclust;

class year (ref='2007') sex (ref='Female') age (ref='15-19') residence (ref='Urban') educ3 (ref='No education') mstatus (ref='Never married') wealthind (ref='Poorest');

*domain variables;

model age1sex*cstatus(**0**)=year/risklimits;

domain age15_64minusne;

**run**;

**proc** **surveyphreg** data=combined1;

weight intwt; strata strata; cluster qclust;

class year (ref='2007') sex (ref='Female') age (ref='15-19') residence (ref='Urban') educ3 (ref='No education') mstatus (ref='Never married') wealthind (ref='Poorest');

*domain variables;

model age1sex*cstatus(**0**)=sex/risklimits;

domain age15_64minusne;

**run**;

**proc** **surveyphreg** data=combined1;

weight intwt; strata strata; cluster qclust;

class year (ref='2007') sex (ref='Female') age (ref='15-19') residence (ref='Urban') educ3 (ref='No education') mstatus (ref='Never married') wealthind (ref='Poorest');

*domain variables;

model age1sex*cstatus(**0**)=age/risklimits;

domain age15_64minusne;

**run**;

**proc** **freq** data=combined1;

tables age25_64minusne*age;

**run**;

**proc** **surveyphreg** data=combined1;

weight intwt; strata strata; cluster qclust;

class year (ref='2007') sex (ref='Female') age (ref='15-19') residence (ref='Urban') educ3 (ref='No education') mstatus (ref='Never married') wealthind (ref='Poorest');

*domain variables;

model age1sex*cstatus(**0**)=residence/risklimits;

domain age15_64minusne;

**run**;

**proc** **surveyphreg** data=combined1;

weight intwt; strata strata; cluster qclust;

class year (ref='2007') sex (ref='Female') age (ref='15-19') residence (ref='Urban') educ3 (ref='No education') mstatus (ref='Never married') wealthind (ref='Poorest');

*domain variables;

model age1sex*cstatus(**0**)=educ3/risklimits;

domain age15_64minusne;

**run**;

**proc** **surveyphreg** data=combined1;

weight intwt; strata strata; cluster qclust;

class year (ref='2007') sex (ref='Female') age (ref='25-29') residence (ref='Urban') educ3 (ref='No education') mstatus (ref='Never married') wealthind (ref='Poorest');

*domain variables;

model age1sex*cstatus(**0**)=mstatus/risklimits;

domain age15_64minusne;

**run**;

**proc** **surveyphreg** data=combined1;

weight intwt; strata strata; cluster qclust;

class year (ref='2007') sex (ref='Female') age (ref='25-29') residence (ref='Urban') educ3 (ref='No education') mstatus (ref='Never married') wealthind (ref='Poorest');

*domain variables;

model age1sex*cstatus(**0**)=wealthind/risklimits;

domain age15_64minusne;

**run**;

* The Adjusted results;

**proc** **surveyphreg** data=combined1;

weight intwt; strata strata; cluster qclust;

class year (ref='2007') sex (ref='Female') age (ref='25-29') residence (ref='Urban') educ3 (ref='No education') mstatus (ref='Never married') wealthind (ref='Poorest');

*domain variables;

model age1sex*cstatus(**0**)=year sex age residence educ3 mstatus wealthind/risklimits;

* domain age25_64minusne;

**run**;

* The Adjusted results-2;

**proc** **surveyphreg** data=combined1;

weight intwt; strata strata; cluster qclust;

class year (ref='2007') sex (ref='Female') age (ref='15-19') residence (ref='Urban') educ3 (ref='No education') mstatus (ref='Never married') wealthind (ref='Poorest');

*domain variables;

model age1sex*cstatus(**0**)=year sex age residence educ3 mstatus/risklimits;

* domain age25_64minusne;

**run**;

**proc** **freq** data=combined1;

table year*sex;

**run**;
