# Supplementary material for: Methods for conducting trends analysis: roadmap for comparing outcomes from three national HIV Population-based household surveys in Kenya (2007, 2012, and 2018)
Source: BMC Public Health. 2022 Jul 13;22:1337. doi: 10.1186/s12889-022-13633-8 (PMC9281165; doi:10.1186/s12889-022-13633-8)
Supplement: Supplementary file 3 — Additional file 3: Supplementary File 3. Proposal for the reweighting of variable in the 2018 Kenya Population-based HIV Impact Assessment (KENPHIA) survey data to match the 2012 Kenya AIDS indicator survey approach. [file 12889_2022_13633_MOESM3_ESM.docx]

**Supplementary File 3. Proposal for the reweighting of variable in the 2018 Kenya Population-based HIV Impact Assessment (KENPHIA) survey data to match the 2012 Kenya AIDS indicator survey approach**

| Step/weights | | Revised KENPHIA weights |
| --- | --- | --- |
| 1 | PSU (EA) Base weight | Inverse of probability of selection of EA  Variable: PSUWT |
| 2 | PSU Nonresponse adjustment | None, however, there are two ineligible EA. The weights of these 2 PSUs is set to 0. |
| 3 | Household initial weight | PSU base weight * inverse of probability of selection of household within EA  HHBWT0=NRPSUWT0 * HH_INPSU_WT; |
| 4 | Unknown eligibility household nonresponse adjustment | HHUNKWT0=HHBWT0 * HHUNK_FACT where HHUNK_FACT is computed within the 19 households within 19 NASCOP/Res (i.e. 10 NASCOP regions by Urban/Rural = 20 – minus rural Nairobi = 19) |
| 5 | Eligible respondent household nonresponse adjustment | HHNRWT0=HHUNKWT0 * HHNR_FACT where HHNR_FACT is computed within the 17 households within 17 NASCOP/Res |
|  |  |  |
| 6 | Person level weights |  |
| 6a | Adults  (15 years old or older) | INDIV_BWT0= HHNRWT0 (all adults were eligible in a household) |
| 6b | Children  (0 <=AGEYEARS<=14) | For households where CHILD_SMPFLG=1. Only children in preselected households  INDIV_BWT0=CH_FACTOR*HHNRWT0  where CH_FACTOR=3;  INDIV_BWT0=0 otherwise |
| 6c | Eligible respondent person nonresponse adjustment | PNR1W0 = INDIV_BWT0 * PNR1F0  Nonresponse adjustments cells are created using NASCOP/Res*SEX  (children/adults are separated for the adjustments) |
| 6d |  | Poststratification by sex and (10) NASCOP regions |
| 7 | Blood test weights |  |
| 7a | Adults/children | Initial weights PNR1W0 |
| 7b | Eligible respondent blood test nonresponse adjustment | PNR2W0= PNR1W0 * PNR2F0  Nonresponse adjustments cells are created using NASCOP/Res*SEX  (children/adults separated for the adjustments) |
| 7c |  | Poststratification by sex and (10) NASCOP regions |
